# Photoacoustic imaging: a unique imaging examination for the assessment of diabetic vascular disease

**DOI:** 10.3389/fcdhc.2025.1651868

**Published:** 2025-11-07

**Authors:** Jieyu Chen, Wenfang Xia

**Affiliations:** Union Hospital of Tongji Medical College of Huazhong University of Science and Technology, Wuhan, China

**Keywords:** photoacoustic imaging, diabetes mellitus, photoacoustic microscopy, diabetic microangiopathy, diabetic foot

## Abstract

As an emerging medical imaging technique, photoacoustic imaging (PAI) holds promise as a significant tool in the field of medical imaging due to its combination of high-resolution optical characteristics and deep penetration acoustic properties. This method non-invasively provides high-contrast biological information imaging and offers substantial advantages in assessing vascular structure and function. The core pathological issue in the clinical diagnosis and treatment of diabetes mellitus is the microvascular and macrovascular complications resulting from hyperglycemia and other factors. However, clinical imaging assessments of vascular lesions are still limited, whereas PAI has demonstrated unique advantages in the early diagnosis, disease monitoring, and therapeutic evaluation of diabetic vascular lesions. PAI not only allows for the calculation of vascular parameters such as vessel diameter and density but also enables real-time monitoring of hemodynamic and blood oxygen saturation dynamics, providing a new approach for the dynamic assessment of diabetic vascular lesions. This article reviews the current research on PAI in diabetic vascular disease, including animal experiments and human studies on microvascular lesions, peripheral vascular disease, diabetic foot, and chronic wounds, showcasing the strengths and limitations of this imaging method to clinicians. This review aims to lay the foundation for further clinical development and application.

## 1 Characteristics and advantages of photoacoustic imaging

**Figure 1:**
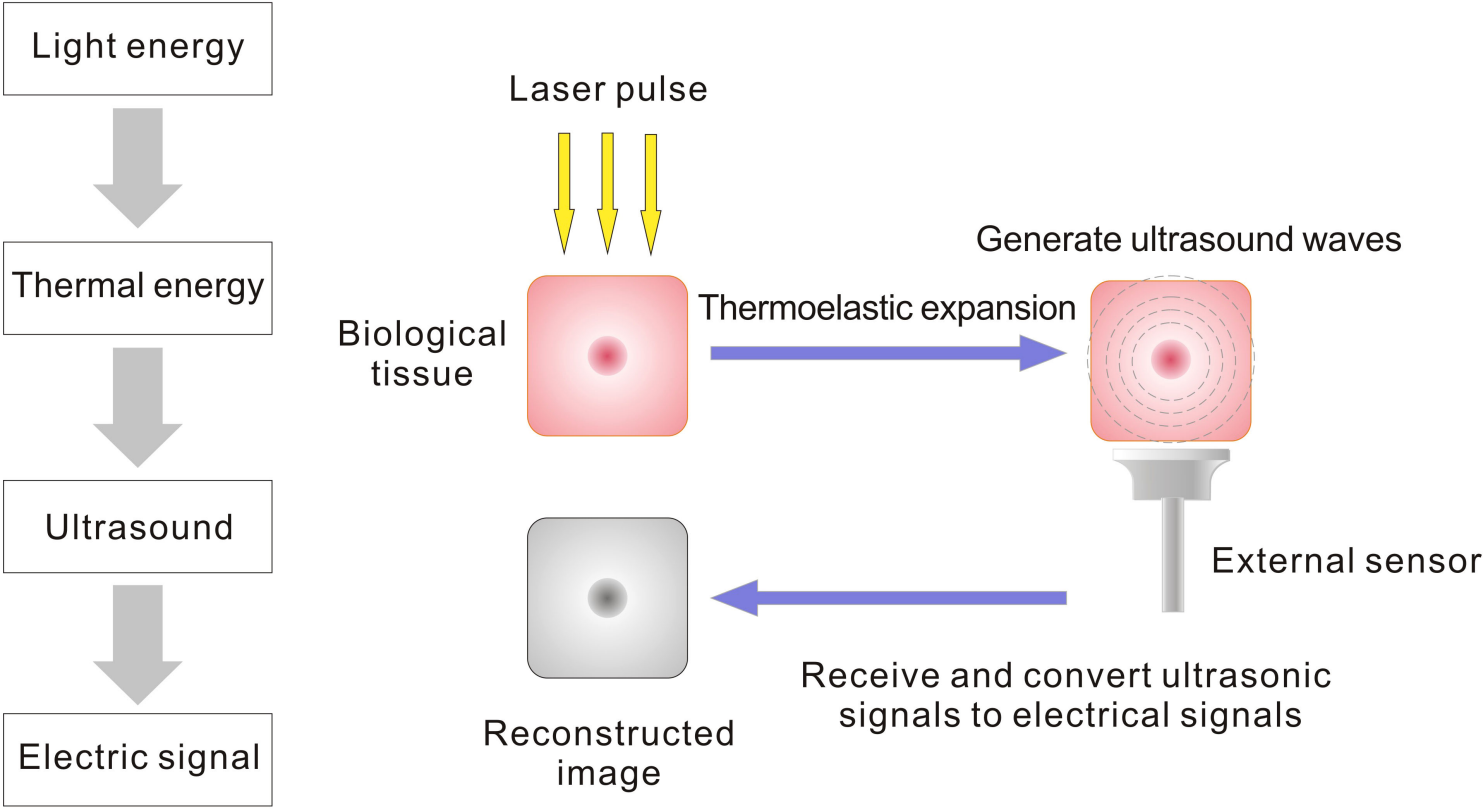
Mechanism of photoacoustic imaging: Laser pulses irradiate biological tissues, which absorb light energy and undergo transient thermoelastic expansion, thereby generating ultrasonic waves. These waves are detected by external sensors (e.g. ultrasound transducers) and reconstructed into images mapping the optical absorption distribution within the tissue.

Since the 1990s, photoacoustic imaging (PAI) has emerged as a novel non-invasive imaging modality and has rapidly developed and been widely applied in the biomedical field. Its principle relies on the detection of acoustic signals generated by biological tissues upon light absorption, enabling reconstruction of optical absorption distribution maps (**Figure 1**). PAI differs from other imaging techniques in several aspects (**Table 1**). For instance, while conventional ultrasonography primarily provides structural information based on acoustic impedance contrasts, PAI leverages optical absorption differences to deliver both anatomical and functional data. Compared to traditional imaging techniques, PAI combines the high selectivity of pure optical tissue imaging with the deep penetration capabilities of pure acoustic tissue imaging. By measuring the optical absorption properties of the imaging area, PAI can achieve high-resolution and high-contrast tissue imaging (5). PAI offers the advantages of being non-ionizing and non-invasive, and it can utilize endogenous contrast agents such as hemoglobin, lipids, melanin, and water, as well as various exogenous contrast agents to obtain information about biological structures, functions, molecules, and dynamics. For example, by detecting hemoglobin’s optical absorption, PAI enables high-quality visualization of the circulatory system, quantifying oxygen saturation and blood flow distribution to reflect tissue metabolism and physiological activity (6). Thus, PAI holds significant potential for vascular assessment in metabolic diseases such as diabetes.

**Table 1:** Comparison of imaging modalities for diabetic angiopathy.

Parameters / Imaging Modalities	Photoacoustic imaging	Ultrasonography	Computed tomography angiography(CTA)	Magnetic resonance angiography(MRA)	Positron emission tomography(PET)	Second near-infrared (NIR-II) window imaging
Spatial Resolution	On the order of tens to hundreds of micrometers	On the order of tens of micrometers to several millimeters (1)	0.2-1mm (2)	0.3-1mm (3)	4-6mm (4)	Its spatial resolution is system-dependent, covering a wide range
Imaging Depth	On the order of several millimeters to several centimeters	Up to several tens of centimeters	Whole body	Whole body	Whole body	On the order of several millimeters to several centimeters
Contrast Agent	Endogenous: hemoglobin, melanin, lipid, water, etc. Exogenous: dyes, metal nanoparticles, etc.	Typically does not require contrast agents;may be used with microbubble contrast agents.	Iodinated Contrast Agents	Gadolinium-Based Contrast Agents	^18^F-FDG, ^18^F-NaF, etc.	Organic fluorophores, inorganic nanoparticles, etc.
Primary Functional Parameters	Vascular morphology, oxygen saturation, hemodynamics, hemoglobin concentration, tissue oxygen metabolic rate, etc.	Vascular Morphology, Hemodynamics	Degree of Stenosis, Hemodynamics, Vessel Wall Characteristics	Degree of Stenosis, Hemodynamics	Standardized Uptake Value, Metabolic Rate, Blood Flow Perfusion	Vascular Morphology, Hemodynamics
Major Advantage	Combines high optical contrast with high resolution, non-invasive and radiation-free, capable of providing multiple functional parameters	Non-invasive and radiation-free, real-time and convenient, relatively low cost	Non-invasive, high-spatial-resolution rapid imaging capable of three-dimensional reconstruction, applicable to vascular imaging of any region throughout the body	Non-invasive, radiation-free, high soft-tissue resolution	Non-invasive whole-body imaging, high sensitivity, precise quantification, provides metabolic and functional tissue information	High spatial resolution, deep tissue penetration, real-time dynamic imaging capability
Main Limitations	Limited penetration depth (particularly for deep large vessel imaging), ongoing clinical translation and standardization efforts, potential interference from local factors such as tissue pigmentation and skin thickness	Penetration depth and resolution are inversely related (trade-off), operator-dependent, limited sensitivity for subtle pathologies	Involves ionizing radiation, risk of contrast-induced nephrotoxicity, and remains limited in depicting subtle vascular pathologies	Longer examination times, risk associated with contrast agents, and limited depiction of microvasculature	Limited spatial resolution, involves radiation exposure, relatively high cost	Potential risks associated with contrast agents, relatively high cost of imaging equipment

PAI technology has developed rapidly, and currently, a variety of imaging modalities have been established to meet different needs. These imaging types include photoacoustic tomography (PAT), photoacoustic microscopy (PAM), photoacoustic mesoscopy (PAMe), among others. The characteristic of photoacoustic tomography is its ability to achieve penetration depths at the centimeter level, with imaging depth far surpassing that of other imaging modalities, making it suitable for structural and functional imaging of internal organs within tissues. The advent of photoacoustic microscopy allows for high-resolution and high-contrast tissue imaging, but its imaging depth is limited. The introduction of photoacoustic mesoscopy fills the gap between photoacoustic macroscopic and microscopic imaging. Its advantage lies in breaking through the limitation of optical diffusion depth while providing images with high spatial resolution, enabling imaging results with both high spatial resolution (less than 100 µm) and a certain penetration depth (1–10 mm) (7).

Subsequently, researchers have combined PAI with other technologies, giving rise to photoacoustic multimodal imaging, which has become a research focus in recent years. PAI can be integrated not only with other imaging modalities such as ultrasound imaging, magnetic resonance imaging, optical coherence tomography, and Near-Infrared-II Imaging but also with molecular probe techniques to form photoacoustic molecular imaging. This combined imaging technology leverages the strengths of different imaging modalities, allowing for simultaneous structural and functional imaging, and achieving comprehensive acquisition of multi-parametric and multi-dimensional information, significantly enhancing the accuracy and functionality of imaging.

Based on the different types of imaging methods and their characteristics mentioned above, photoacoustic imaging can play a role in the field of diabetes mellitus, where vascular lesions are a core concern. For instance, photoacoustic tomography can be applied to vascular imaging (8). Photoacoustic microscopy is suitable for high-resolution imaging at a millimeter depth, including skin microvascular imaging (9), and is of great significance in the study of subcellular and cellular microstructural tissues. Xiao et al. (10) developed a novel fluorescence and photoacoustic dual-modality probe to investigate diabetes-induced hepatic injury and the resulting microstructural and morphological alterations in liver tissue. Yang et al. (11) combined ultrasound with PAT for the study of diabetic foot, enabling more reliable non-invasive quantification of vascular parameters in the foot.

The aforementioned research demonstrates that photoacoustic imaging offers a novel evaluation method for the diagnosis and treatment of diseases such as diabetic vascular lesions. This article reviews the current animal experiments and human studies on microvascular lesions, peripheral vascular lesions, diabetic foot, and chronic wounds, aiming to further clarify the advantages of PAI in the field of diabetes and lay a theoretical foundation for the development and clinical application of PAI.

## 2 Evaluation value of photoacoustic imaging in diabetic microangiopathy

The characteristic changes of diabetic microvascular lesions include thickening of the microvascular basement membrane and microcirculatory disturbances. However, the use of traditional histological methods to observe these lesions may alter the morphology of the samples, thereby affecting the accurate assessment of physiological characteristics. Existing examinations have limited capacity for evaluating diabetic microvascular complications, but PAI, as a non-invasive technique, offers a unique perspective for observing microvasculature within the body. Due to the different optical absorption coefficients of normal tissues and red blood cells in blood for specific wavelengths of laser light, PAI can reflect the condition of microvascular lesions by capturing the absorption of light within tissues and the acoustic waves generated by the tissue (12), which is of great significance for assessing the severity of diabetes.

Despite the aforementioned characteristics of PAI technology, its penetration depth remains limited. Consequently, current research on the application of PAI in microvascular lesions primarily focuses on the microvasculature of the skin, eyes, and skeletal muscle. These studies provide a foundation for further exploration of PAI in the diagnosis of diabetic microvascular lesions and are expected to offer more precise diagnostic and therapeutic strategies for patients with diabetes in the future.

### 2.1 Skin microvessel

**Figure 2:**
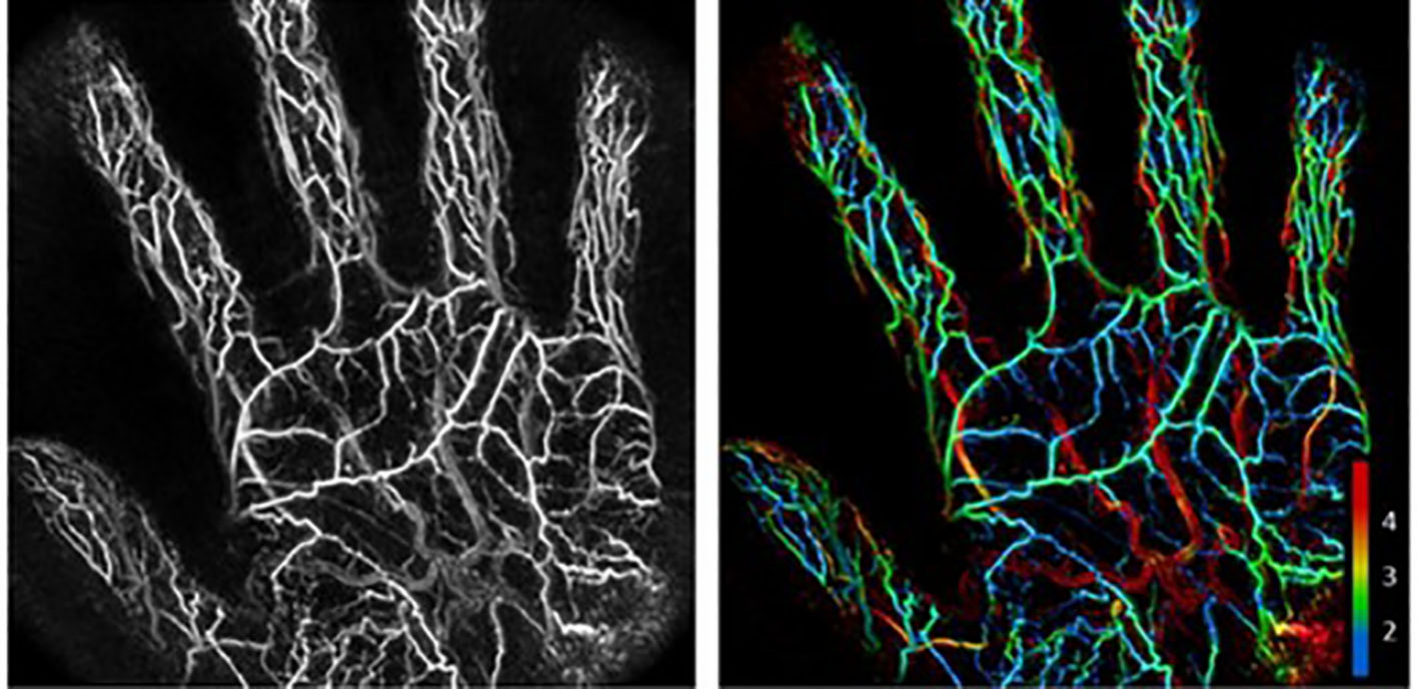
A maximum intensity projected photoacoustic image of the palm of an individual’s hand alongside the same image with vessel depth represented through color coding. Numbering on the bottom right of the figure represents vessel depth in mm. Copyright Notice: Used with permission of IOP Publishing, Ltd, from Microvascular imaging of the skin, Deegan AJ, Wang RK, 64, ISSN:1361-6560,2019; permission conveyed through Copyright Clearance Center, Inc.

Cutaneous microvasculature not only provides structural support but also plays a key role in defense and immune responses. Diabetes mellitus, however, can damage the skin microvascular system, thereby compromising the skin’s barrier protective function. At present, a variety of techniques have been employed for the assessment of skin microvessels, including laser Doppler flowmetry (13), nailfold capillaroscopy (14, 15), dermatoscopy, Doppler ultrasound, laser speckle contrast imaging (LSCI), optical coherence tomography, and spatial frequency domain imaging (16). Among these, LSCI assesses blood flow velocity by analyzing dynamic fluctuations of laser speckle patterns. While it offers advantages such as full-field imaging and high temporal-spatial resolution, it remains susceptible to tissue motion and illumination distance, with additional limitations including limited penetration depth. Consequently, it is primarily suitable for evaluating cutaneous microcirculatory dysfunction (17). These techniques primarily focus on observing the structure and morphology of microvessels but lack imaging assessments related to the metabolic aspects of diabetic microvascular lesions, failing to reveal the associations between microvessels and metabolism, blood oxygenation, inflammation, and other factors. Nonetheless, some current research has begun to explore this aspect. PAI has garnered attention due to its non-invasive nature, high selectivity, and deep penetration, offering new possibilities for the exploration of skin microvessels (**Figure 2**).

PAI can correlate with vascular parameters and metabolic indicators, thereby assessing microvascular lesions and inflammatory states in the skin. The study by Yao et al. (18) utilized photoacoustic microscopy (PAM), which is capable of not only imaging the skin of healthy mice but also calculating vascular morphometric parameters such as vessel diameter distribution, microvascular density, vascular tortuosity, and fractal dimension, demonstrating the applicability of PAI in skin microvessel monitoring. Favazza et al. (9) used PAM to obtain vascular images of the skin from the forearms and palms of healthy volunteers, confirming the potential of PAI in characterizing the structure and function of human skin microvascular networks. Krumholz et al. (19) further focused on type 1 diabetic mice and used PAM to monitor the effects of varying blood glucose levels on vascular morphology and function. Their research linked changes in blood glucose metabolism to skin vascular imaging, confirming that PAI can effectively assess microvascular damage induced by disorders of glucose metabolism.

In addition to photoacoustic microscopy, photoacoustic mesoscopy can also be applied to skin microvascular imaging. The study by Karlas et al. (20) demonstrated that photoacoustic mesoscopy can reflect the characteristic changes in the skin of individuals with diabetes. By using a raster-scanning photoacoustic mesoscope to extract images from the leg skin of healthy individuals and diabetic patients, the researchers found that the skin characteristics changed at different stages of diabetes, primarily in the dermis and the epidermis/subpapillary vascular plexus layers. Photoacoustic mesoscopy offers high-contrast imaging of vascular structures and high-resolution three-dimensional visualization of the epidermis and dermis, as well as directly associating glucose metabolism with skin imaging. Moreover, skin microvascular lesions often occur in the early stages of diabetes, possibly preceding microvascular complications in other organs and also preceding macrovascular complications and marked hyperglycemia (blood glucose levels ≥200 mg dL^-1^). Therefore, photoacoustic mesoscopy is of great importance for the prevention and control of early microvascular lesions in diabetes.

### 2.2 Ocular microvessels

Diabetic retinopathy (DR) is the most common retinal vascular disease and one of the leading causes of blindness in individuals over the age of 40. The fundamental pathological process of DR involves damage to microvascular cells, leading to microvascular dilation, microaneurysms, leakage, and subsequently to microvascular occlusion and the formation of non-perfused areas. This results in retinal ischemia and hypoxia, ultimately progressing to proliferative lesions. Therefore, it is particularly important to adopt appropriate diagnostic methods and to manage DR early in its course.

Currently, clinical ophthalmic imaging predominantly utilizes optical imaging techniques. However, these technologies often face challenges such as invasiveness or depth limitations, preventing effective non-invasive visualization of deep structures like the choroid. These optical imaging modalities include optical coherence tomography, optical coherence tomography angiography, scanning laser ophthalmoscopy, adaptive optics, fundus autofluorescence, and molecular imaging. Although some of these imaging technologies have evolved to achieve high spatial resolution, they still suffer from limited penetration. Moreover, most imaging techniques primarily provide anatomical information, with relatively little physiological information (21).

PAI is capable of providing anatomical and functional features of the eye, particularly excelling in visualizing blood vessels. The photoacoustic funduscope is a newly developed instrument based on PAI for examining the ocular microcirculation. Compared to traditional light-based imaging systems, it offers greater tissue penetration depth. This technology cleverly utilizes hemoglobin and melanin, the two dominant natural chromophores in retinal optical absorption, allowing for the acquisition of structural and functional information without the need for exogenous contrast agents (5). Moreover, PAM can achieve an axial resolution as high as 2.5 μm and a lateral resolution as high as 0.4-0.7 μm, enabling the diagnosis and monitoring of minute intracular structures and cells (22). However, the resolution decreases with increasing imaging depth.

Multimodal imaging has demonstrated great potential in the visualization of diabetic retinopathy. By integrating the advantages of different imaging modalities, PAI can more accurately assess the structure and function of the retina. This technology aids in a deeper understanding of the pathogenesis and pathological processes of the disease, providing a scientific basis for future therapeutic strategies. Nguyen et al. (23) combined photoacoustic microscopy (PAM) with spectral-domain optical coherence tomography (SD-OCT) to observe rabbit retinal neovascularization (RNV). This system clearly characterized the location and morphology of individual RNV, capable of displaying high-resolution hemoglobin optical absorption as well as retinal and choroidal vascular imaging. The research by Du (24) further expanded this field. They established a dual-modal imaging system combining PAM and SD-OCT for *in vivo* fundus imaging of rats with choroidal neovascularization, and incorporated nanoprobes as exogenous contrast agents to enhance imaging, thereby more comprehensively obtaining information on choroidal vasculature. These studies have validated the feasibility of photoacoustic and multimodal imaging systems for *in vivo* ocular imaging, expanded the diagnostic approaches for diabetic retinopathy, and provided new insights and tools for the diagnosis and treatment of related diseases.

### 2.3 Skeletal muscle microvessels

Diabetes can lead to impairments in the structure, function, and metabolic capacity of skeletal muscle, clinically manifesting as reduced muscle mass, muscle weakness, and decreased exercise capacity, a condition referred to as diabetic myopathy (25). The microvascular lesions in skeletal muscle are a significant factor contributing to changes in muscle mass and quality in patients with diabetes. Impaired function of the skeletal muscle microvasculature results in insufficient nutritional support for skeletal muscle cells, thereby affecting the normal metabolism and function of muscle cells. However, there are currently limited methods for evaluating skeletal muscle microvascular perfusion, each with its own limitations. For instance, contrast-enhanced ultrasound has been used in studies to assess skeletal muscle microcirculation in healthy individuals and patients with diabetes (26, 27), but low-power contrast-enhanced ultrasound imaging produces a relatively low signal-to-noise ratio, and while higher power can yield a stronger signal, it may lead to microbubble destruction (28); MR perfusion imaging (29) is time-consuming and costly, precluding its use as a routine examination; 1-methylxanthine, a substrate for xanthine oxidase, can be measured by liquid chromatography to quantify microvascular perfusion, but its application is limited due to the low activity of xanthine oxidase in the human skeletal muscle microcirculation (30, 31).

In contrast, PAI offers advantages in skeletal muscle microvascular imaging with high contrast, high penetration, allowing for noninvasive functional, metabolic, and histological imaging applications. Loai et al. (32) utilized a high-frequency ultrasound photoacoustic imaging system to image a type 2 diabetic rat model and identified a significant reduction in leg skeletal muscle perfusion, aiding in the understanding of the manifestations of diabetic skeletal muscle microvascular dysfunction. Furthermore, the research group (33) discovered that early reductions in leg skeletal muscle perfusion in diabetic cardiomyopathy were associated with gender-related microvascular abnormalities, with female diabetic rats exhibiting a significantly delayed decrease in skeletal muscle tissue oxygen saturation compared to male diabetic rats. This indicates that photoacoustic imaging technology can be used to assess microvascular perfusion in diabetic myopathy and to reveal gender differences. However, the researchers noted that the sensitivity of using PAI to indirectly measure perfusion may not be very high, and significant changes in oxygen metabolism, possibly indicative of more severe microvascular complications, may be required to meet the sensitivity threshold for photoacoustic imaging, which could result in the missed detection of perfusion impairment in the legs of female diabetic rats at an early stage.

Despite its limitations, the potential of photoacoustic imaging technology in assessing microvascular perfusion in diabetic myopathy remains noteworthy, and it is anticipated that future technological innovations will enhance its sensitivity and clinical applicability.

## 3 The value of photoacoustic imaging in the assessment of diabetic peripheral vascular lesions

Peripheral vascular disease significantly impacts the quality of life in diabetic patients. Due to insufficient blood supply in the peripheral vessels, the nutrient supply required for tissue metabolism decreases, leading to local symptoms such as pain, cold sensation, numbness, and ulceration or gangrene. Current research on peripheral vascular lesions includes local peripheral vasculature, diabetic foot, and chronic wounds. The following section will focus on the evaluative value of photoacoustic imaging in these diabetes-related peripheral vascular lesions.

### 3.1 Local peripheral vasculature

Existing methods for assessing local peripheral vascular function have certain limitations: Ultrasound imaging is non-invasive and convenient, but lacks sensitivity for small vessels; computed tomography angiography requires the use of nephrotoxic contrast agents, which is problematic as diabetes patients often have kidney damage; intravascular ultrasound and optical coherence tomography are invasive and their application is limited to large arteries; magnetic resonance angiography can provide details of the circulation in the upper and lower extremities, but does not offer direct perfusion information and still requires the use of nephrotoxic contrast agents; diffuse optical tomography can provide dynamic changes of deep vessels, but has relatively low resolution; laser Doppler imaging is simple to use and can monitor perfusion of foot vessels, but is limited to superficial tissues. In the realm of emerging imaging technologies, diffuse speckle contrast analysis (DSCA), an advanced development based on speckle techniques in recent years, has overcome the depth limitations of traditional LSCI. It enables blood flow measurements in deep tissues and can extract tissue perfusion indices for quantification, showing potential in the assessment of diabetic foot perfusion. Nevertheless, DSCA remains sensitive to motion artifacts and requires further optimization of probe mechanical stability (34). In parallel, NIR-II imaging utilizes the second near-infrared window (approximately 900–1880 nm) to achieve high signal-to-background ratio and high-resolution vascular visualization, with high sensitivity to perfusion changes in early-stage pathologies. Studies have demonstrated (35) that NIR-II imaging can acutely detect microcirculatory perfusion impairments caused by arterial plaques even during the preclinical stages of peripheral arterial disease. In research by Wang et al. (36), NIR-II imaging clearly revealed vascular truncation planes, collateral circulation development, and quantitatively evaluated perfusion recovery at different time points. While NIR-II imaging provides a novel perspective for early warning and intervention of diabetic vascular complications, its dependence on contrast agents currently limits broad clinical application.

PAI, as a novel non-destructive imaging technology, possesses the capability to monitor and assess peripheral vasculature. Joongho et al. (37) utilized photoacoustic microscopy to non-invasively monitor morphological changes in the rat limb vessels during rapid increases in blood glucose levels and identified their correlation with acute hyperglycemia. Jinge Yang et al. (38) extended the research to human studies, employing photoacoustic tomography to monitor vascular dynamics during lower limb vascular stimulation, demonstrating hemodynamic responses observed within the vessels and assessing the magnitude and time parameters of reactive hyperemia following arterial occlusion, confirming the decline in vascular function with aging. Wu Man’s study (39) further included observations in diabetic patients. In this study, PAT was used to image the finger vessels of two groups of volunteers (healthy and diabetic) and to monitor hemodynamic changes before and after vascular occlusion, uncovering imaging evidence of diminished vascular function in diabetic patients. These studies indicate that PAI is feasible in assessing limb vascular function and provides a new tool for the prevention and control of diabetic limb vascular complications.

**Figure 3:**
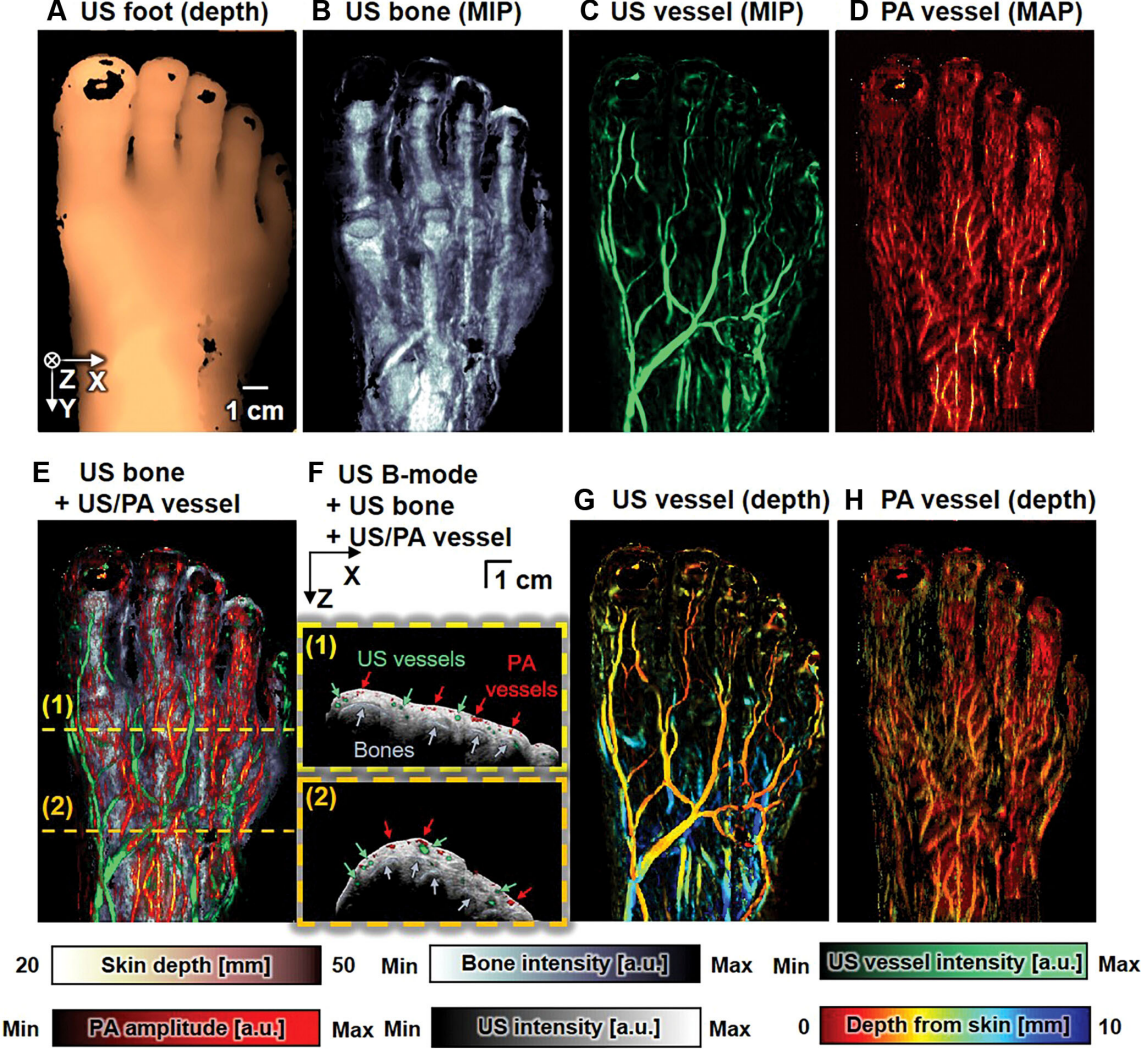
Multistructural noncontrast photoacoustic (PA)/US images obtained in a healthy 32-year-old male volunteer’s foot. **(A)** US skin image based on the two-dimensional depth profile of skin, **(B)** US maximum intensity projection (MIP) bone image, **(C)** US MIP vessel image, and **(D)** PA maximum amplitude projection (MAP) vessel image. **(E)** Multistructural overlay of B, C, and D **(F)** Overlaid cross-sectional images: conventional US B-mode, US bone, US vessel, and PA vessel. Depth-colored **(G)** US vessel and **(H)** PA vessel images. a.u. = arbitrary units, max = maximum, min = minimum. Copyright Notice: Used with permission of RADIOLOGICAL SOCIETY OF NORTH AMERICAN, Ltd, from Three-dimensional Multistructural Quantitative Photoacoustic and US Imaging of Human Feet in Vivo, Choi W, Park EY, Jeon S, Yang Y, Park B, Ahn J, Cho S, Lee C, Seo DK, Cho JH, Kim C, 303, ISSN:1527-1315,2022; permission conveyed through Copyright Clearance Center, Inc.

Because of the characteristics and advantages of photoacoustic imaging in peripheral vascular evaluation, researchers have used it specifically for the evaluation of diabetic foot, and most of them are photoacoustic and multimodal imaging. In the study by Choi et al. (40), a photoacoustic/ultrasound imaging system integrated with a foot scanner was able to clearly visualize the structures of blood vessels, bones, and skin without the use of contrast agents, and reliably quantify foot vascular parameters non-invasively, providing functional information such as total hemoglobin concentration, hemoglobin oxygen saturation, vascular density, and vessel depth (**Figure 3**). Jinge Yang et al.’s research (11) included observations of diabetic foot, where they used a photoacoustic tomography system based on a concave ultrasound sensor array to image the dorsum of the foot in both diabetic and healthy subjects. They observed lower levels of oxygen saturation in diabetic patients compared to healthy subjects, and their peripheral blood exhibited a distinct hemodynamic response.

The aforementioned studies indicate that photoacoustic imaging technology has certain applicability in detecting vascular dysfunction in diabetic foot and assessing therapeutic efficacy. However, these studies also highlight some limitations, such as the limited light penetration due to the presence of bone structures, and the potential for non-uniform sensitivity in the PAT obtained from the laser irradiation method on the side of the sensor array. Future research could visualize the plantar aspect by adding another imaging probe at the bottom. Additionally, the issue of non-uniform sensitivity could be addressed by using PA coupling pads with light diffusion or quantitative PAT methods, further optimizing the application of photoacoustic imaging technology in the diagnosis of diabetic foot.

### 3.2 Chronic wound

Chronic wounds, including diabetic ulcers and pressure ulcers, represent a significant challenge in the healthcare field. Pressure ulcers can be categorized according to the depth and severity of the injury into stages of erythema, inflammatory infiltration, shallow ulceration, and necrotic ulceration. The necrosis of ulcers can be attributed to reduced blood flow under sustained pressure, leading to vascular dysfunction of the tissue. Both pressure ulcers and diabetic foot ulcers result in tissue vascular dysfunction, but this dysregulation is often difficult to detect in the early stages.

Currently, imaging modalities for chronic wound examination include ultrasound, computed tomography, magnetic resonance imaging, optical coherence tomography, and others. However, some of these imaging modalities are challenging in assessing early-stage damage in chronic wounds. The advantage of PAI lies in its capability to detect early lesions, non-invasively observing photoacoustic intensity changes associated with early tissue damage, thus preventing further ulcer progression and epithelial barrier disruption. In a murine pressure ulcer model, Hariri et al. (41) evaluated alterations in photoacoustic signals and injury depth. The results indicated that photoacoustic imaging can differentiate between different stages of pressure ulcers and monitor the healing process. Nevertheless, the study also highlighted limitations of photoacoustic imaging, such as difficulties in imaging through bone and the possibility that photoacoustic signals from deep tissues may not fully represent the severity of the ulcer.

Remarkably, PAI also plays a role in assessing the efficacy of treatments for chronic wounds. For instance, hyperbaric oxygen therapy is a common treatment for chronic wounds aimed at improving blood and tissue oxygenation, promoting angiogenesis, and accelerating wound healing. In a study monitoring chronic wound healing under hyperbaric oxygen therapy, Mantri et al. (42) employed photoacoustic imaging (PAI) to track changes in hemoglobin oxygen saturation and perfusion, generating 3D maps of forearm vascular oxygenation that validated the correlation between PA signals and hemodynamic parameters. Additionally, there was a significant increase in radial artery oxygen saturation after hyperbaric oxygen therapy, indicating that PAI can be used to evaluate the efficacy of hyperbaric oxygen therapy for chronic wounds. Nevertheless, limitations of photoacoustic imaging due to skin thickness and pigmentation (melanin concentration) should be noted. Variations in skin thickness and pigmentation may affect the penetration of light into tissue and the accuracy of measurement results. Subjects with darker skin may experience increased light absorption at the skin surface, leading to reduced sO_2_ readings, decreased penetration depth, and blurred imaging of the underlying vascular system.

In summary, photoacoustic imaging holds significant application value in the diagnosis and treatment monitoring of chronic wounds, but further research is needed to overcome its limitations and to enhance its accuracy in clinical applications. With the continuous development and optimization of the technology, photoacoustic imaging is expected to become an important tool for assessing chronic wounds in the future.

## 4 Limitations of photoacoustic imaging

Although PAI technology exhibits tremendous potential in the field of biomedical imaging, there are currently several limitations that need to be addressed.

1. Penetration depth is limited. Despite PAI offering superior penetration depth compared to conventional optical methods, its maximum penetration depth is still limited, typically reaching only several centimeters, which restricts its application in imaging large organs or even the entire body. Furthermore, due to interference factors such as the presence of bony structures, PAI is unable to perform imaging of large organs or whole-body imaging; thus, it is currently primarily used for more superficial areas such as animal models and limb extremities.
2. Spatial resolution is limited. To a certain extent, the spatial structural resolution of PAI is inferior to that of three-dimensional imaging techniques such as computed tomography and magnetic resonance imaging, primarily due to its limitation to one-dimensional or two-dimensional detection. Moreover, the resolution of PAI decreases with increasing imaging depth due to optical and acoustic attenuation, leading to a reduced signal-to-noise ratio in deeper tissues (43), and consequently, the reliability of the information conveyed by photoacoustic signals from deep tissues also diminishes. However, when PAI is combined with high-resolution imaging modalities such as CT, its resolving power can be significantly enhanced.
3. Detection sensitivity is suboptimal. PAI has limited detection efficacy for certain substances at low concentrations; moreover, the sensitivity of indirectly measuring perfusion changes through blood oxygen saturation may not be very high and may only meet the sensitivity threshold of PAI in more severe conditions (33), which could impede its use for detecting early tissue damage in some cases. Therefore, the development of more characteristic concentration detection methods is needed to address this limitation.
4. Dependence on coupling media. Most PAM requires a coupling medium, such as water or ultrasound gel, which means that photoacoustic ocular examinations necessitate physical contact with the eye, potentially causing discomfort to the patient. This also implies that the application of PAM may be restricted in certain settings (44).

Overall, despite the significant advantages of PAI technology in the field of biomedical imaging, it still faces numerous challenges in clinical application. Furthermore, in its later clinical use, it is necessary to overcome difficulties such as long imaging time and slow imaging speed (45), to enhance its cost-effectiveness and portability (46, 47), and to reduce the interference from factors such as skin thickness and pigmentation. In future research, it will be essential to further optimize imaging techniques and develop new image processing and analysis methods to achieve widespread application of PAI technology.

## 5 Summary

PAI, as a non-ionizing and non-invasive imaging technology, is increasingly becoming a research focus due to its characteristics such as high resolution, deep penetration depth, high sensitivity, and high contrast. Currently, there are limited trials of PAI in diabetic animals or human subjects, and it is still in the early stages of development. In terms of vascular parameter detection, hemodynamic monitoring, and tissue injury exploration, PAI has demonstrated its ability to reflect the impact of metabolic factors on vascular lesions more effectively than traditional examinations.

Research on a variety of metabolism-related PAI contrast agents has opened new avenues for its development. In addition to hemoglobin as an endogenous contrast agent, there are several endogenous and exogenous contrast agents available for use with PAI. For instance, when lipids are used as endogenous contrast agents, PAI can be employed to non-invasively assess the metabolism of brown adipose tissue in living animals (48) and detect differences in brown adipose tissue between healthy and diabetic tissues (49, 50). Moreover, since blood glucose concentrations can affect photoacoustic signals, PAI can also be used for non-invasive glucose measurement (51–54). When combined with photoacoustic probes as exogenous contrast agents, PAI can assist in the visualization of pharmaceutical research and diagnostic treatment, such as studying the dynamics of injection sites of near-infrared dye-labeled insulin preparations (55), validating the targeting of melanin-based targeted nanodrugs for the treatment of diabetic nephropathy (56), and developing novel probes for detecting diabetic liver injury (10), among other applications.

**Figure 4:**
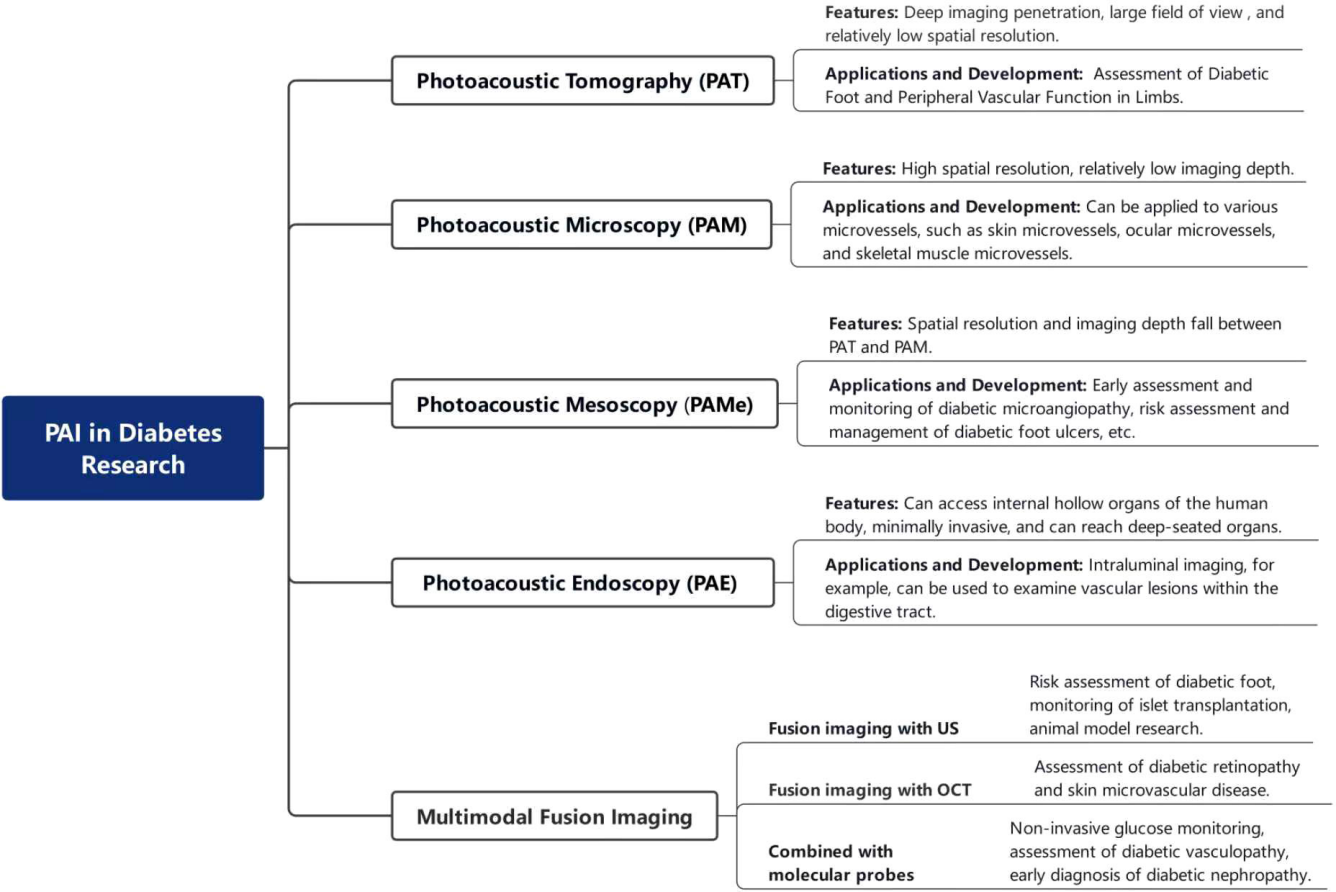
PAI in diabetes research.

The research and development of PAI contrast agents, the integration of multi-modal imaging, and the combination with diagnostic and therapeutic visualization will contribute to the further advancement of PAI in imaging diabetes and its complications, and will provide support for future diabetes research. With continuous technological progress and innovation, PAI is expected to play an even more significant role in the field of diabetes diagnosis and treatment in the future (**Figure 4**).
